# MIGRATION OVER THE LIFE COURSE AND LATER-LIFE DEPRESSION IN CONTEMPORARY CHINA

**DOI:** 10.1093/geroni/igad104.2151

**Published:** 2023-12-21

**Authors:** Nan Zhang

**Affiliations:** University of Manchester, Manchester, England, United Kingdom

## Abstract

Migrating between rural and urban areas over the life course profoundly shapes the conditions of later life. In the Chinese context, living in urban areas with an urban Hukou is associated with socioeconomic advantage. This study is among the first attempt to investigate how migration into urban areas in China is related to these processes and the association with risk of depression in later life by focusing on the timing and the type of migration (rural-urban residential mobility and/or institutional transition of Hukou status) of migration. Using data from China Health and Retirement Longitudinal Study, we found strong associations between migration over the life course and risk of depression in later life in China. The timing and type of migration appears to play an important role. In-situ urbanisation is associated with lower depression scores in later life, and these effects are greater for in-situ urbanisation occurring in middle age compared with young adulthood. Forced urban-rural migration is associated with improved mental wellbeing. Formal social protection, particularly having a private pension, contributes substantially to the mental health advantage of social groups with an urban Hukou. Having an urban Hukou origin has an independent protective role in shaping mental wellbeing in later life in China, potentially partly due to the entitlement to a private pension attached to this status. When informal support has weakened in contemporary China, enhanced formal social protection in the form of adequate pensions should be put in place to mitigate structural inequalities associated with migration in old age.

